# Measuring Accurate Body Parameters of Dressed Humans with Large-Scale Motion Using a Kinect Sensor

**DOI:** 10.3390/s130911362

**Published:** 2013-08-26

**Authors:** Huanghao Xu, Yao Yu, Yu Zhou, Yang Li, Sidan Du

**Affiliations:** School of Electronic Science and Engineering, Nanjing University, Nanjing 210046, China; E-Mails: huanghaoxyu@gmail.com (H.X.); yogo@nju.edu.cn (Y.L.)

**Keywords:** human body measurement, motion capture, human body modeling, Microsoft Kinect

## Abstract

Non-contact human body measurement plays an important role in surveillance, physical healthcare, on-line business and virtual fitting. Current methods for measuring the human body without physical contact usually cannot handle humans wearing clothes, which limits their applicability in public environments. In this paper, we propose an effective solution that can measure accurate parameters of the human body with large-scale motion from a Kinect sensor, assuming that the people are wearing clothes. Because motion can drive clothes attached to the human body loosely or tightly, we adopt a space-time analysis to mine the information across the posture variations. Using this information, we recover the human body, regardless of the effect of clothes, and measure the human body parameters accurately. Experimental results show that our system can perform more accurate parameter estimation on the human body than state-of-the-art methods.

## 1. Introduction

Non-contact human body measurement plays an important role in surveillance, physical healthcare, on-line business and virtual fitting. Usually, we must acquire human body models before biometric measurements. Laser range scanners can provide human body reconstruction, which can be used for accurate biometric measurements. However, laser range scanners cost from $40,000 to $500,000 and require people to wear tight clothing or almost no clothes. Therefore, laser range scanners cannot be an everyday choice for human body measurement in the short term. Recently, marker-less multi-view systems [1–4] have become more and more proficient at acquiring human body models because of the efforts of researchers; however, these solutions always take too much space and are difficult to set up. At the same time, commercially-produced multi-view systems do not have a considerable price advantage. With an in-depth study on the accuracy of the Microsoft Kinect sensor [5], monocular systems have become an appropriate choice. Weiss *et al.* [6] obtained four different views of depth maps and RGBimages from the Kinect sensor, and estimated a consistent model from them. Their work provides a simple, but effective, solution for home 3D body scans, but their solution cannot handle large-scale motion and the condition of people wearing clothes. Thus, it is inconvenient to use their proposed methods in a public environment. Cui *et al.* [7] estimated a model of good quality from 12 views of depth maps and RGB images, but their solution also suffers from large-scale motion. Therefore, human body measurement with large-scale motion is still an open problem.

When measuring the human body with large-scale motion, the first priority is recovering accurate pose parameters. Recently, the technology of motion capture [8], especially monocular motion capture [9,10], has seen rapid development. Shotton *et al.* [11] formulated 3D pose detection as a per-pixel classification problem. This solution belongs to bottom-up technology in computer vision, and it can give a coarse estimate of human motion without any initialization. Ganapathi *et al.* [12] derived an efficient filtering algorithm for tracking human poses. Furthermore, Wei *et al.* [13] formulated the 3D pose tracking as a maximum a *posterior* (MAP) problem. However, current technology in monocular human motion capture often gives poor estimations of the motion of roll, which leads to inaccurate reconstruction of human limbs and heads. One of the contributions of our work is extending current monocular motion capture technology to acquire a more accurate SCAPEmodel [14] with large-scale motion.

In addition to the large-scale motion, the effect of clothes is another challenge for accurate body measurement. The KinectAvatar [7] can partly capture the average geometry of clothing, but it cannot measure accurate parameters of the human body under the clothes. DRAPE[15] is a good method for animating realistic clothing on bodies. However, it does not give an inverse model to mitigate the effect of the clothes when measuring the human body. Nils Hasler *et al.* [16] present a novel method for estimating the body shape of dressed humans, but their method is based on a setup of synchronous multiple RGB cameras in such a way that human interaction is required. Inspired by Non-Rigid Structure from Motion (NRSfM) technology [17–20], we resort to the cue of large-scale motion over time. When people wearing everyday clothes, e.g., T-shirts, shirts and spring blouses, conduct large-scale motion in front of the sensor, some part of the clothes can be close to the human body. Based on this observation, we collect all of the information over time and recover a model that is closest to the human body.

In this paper, we present a novel approach to measuring the human body with large-scale motion from a single Kinect sensor, regardless of whether people wear clothes or not. In our approach, we combine pose detection with pose tracking as a multi-layer filter to estimate accurate pose parameters from the monocular Kinect sensor. Then, we estimate a consistent model of people who are engaged in large-scale motion. Afterward, we mitigate the effect of clothes through space-time analysis, and we measure the body parameters accurately from the human model.

In summary, our contributions are: (1) A multi-layer framework for accurate human motion capture in the monocular, noisy and low-resolution condition. The combination of pose detection, pose tracking and failure detection achieves a fully automatic process of human motion estimation. (2) The application of a space-time analysis to mitigate the effect of clothes, which makes it possible to apply our system non-intrusively in public environments.

## 2. Overview

**Figure 1.**
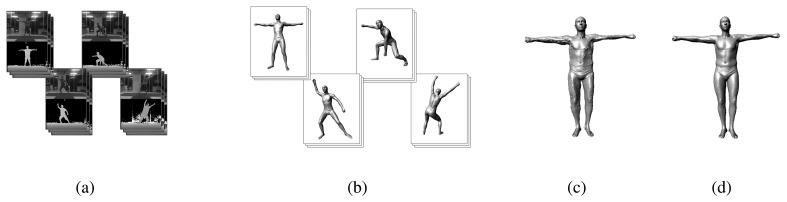
Pipeline of our approach: (**a**) the input RGB images and depth maps from Kinect; (**b**) different poses recovered for every frame; (**c**) the spatial-temporal average model reconstructed across all the frames; (**d**) the model after mitigation of the clothes effect.

In this paper, we present a system for measuring humans wearing clothes with large-scale motion. At first, a video sequence of people acting in diverse poses is captured by a monocular Kinect sensor (Figure 1a). Afterward, we recover the pose for every frame in the sequence following a fully automatic multi-layer framework (Figure 1b). The multi-layer framework is composed of a pose-detection module, a pose-tracking module (Section 3.1) and a failure-detection module (Section 3.2). For pose detection, we use a method that is similar to [11]. In contrast to [11], we use the SCAPE model to construct the synthetic training database. Our database has approximately 50,000 poses and body shapes of 300 different individuals. For the pose tracking, we extend the method proposed by Wei *et al.* [13] from the cylinder-like model to the SCAPE model. Pose tracking, in which we adopt both the cue from the RGB image and the silhouette constraint, provides accurate pose estimation for every frame, while pose detection provides coarse pose estimation for the initialization of the first frame and recovery from the failed pose tracking.

After the motion capture step, we estimate the models according to the depth maps for different poses. Because we have recovered accurate pose parameters for every frame, we can transform all of the models of the different poses into a standard pose, and then, a spatial-temporal average model can be reconstructed (Section 4.2, Figure 1c). To mitigate the effect of clothes, a space-time analysis is performed after the average model reconstruction (Section 4.3, Figure 1d). Then, we measure human body parameters with less effect from the clothes.

## 3. Motion Capture

**Figure 2.**
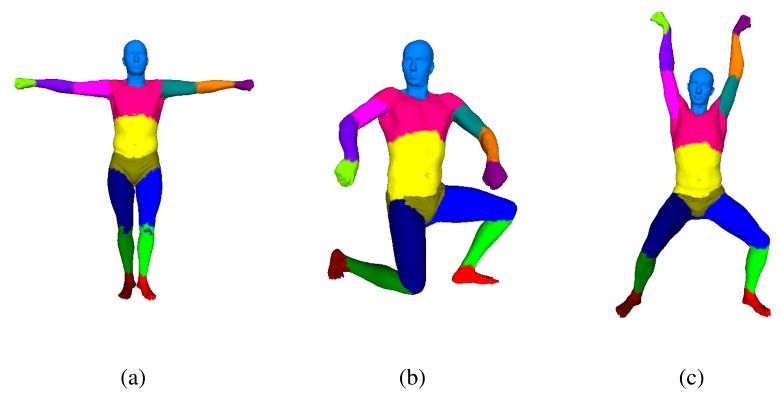
(**a**) Template SCAPE model with 16 color-coded parts and (**b**),(**c**) SCAPE model of different poses.

In our overall framework, we solve human pose parameters and shape parameters separately. To do so, we adopt the SCAPE model, which is a parametric method of modeling human bodies that factors the complex non-rigid deformations induced by both pose and shape variation and is learned from a database of several hundred laser scans. The database that we have used to train the SCAPE model is from [21]. In this section, we will introduce our motion capture module based on the SCAPE model. We approximate human-body geometry with 16 rigid parts (Figure 2), and we use 36 degrees of freedom (DoF) to describe people's different poses. Our motion capture step follows a multi-layer framework: the pose-detection module provides coarse pose estimation for initialization, and the pose-tracking module reconstructs human poses from successive frames. Because our pose-tracking module can fall into failure sometimes, a failure-detection module is used to detect failed pose tracking, and we call the pose-detection module to re-initialize the motion capture system.

### 3.1. Preliminaries

In the following parts of this section, we will represent the pose parameters that are estimated for the human body as **q** ∈ *R*^36^, where the first six degrees of freedom represent the absolute root position and orientation, and the other degrees of freedom represent the relative joint rotations. These joints are the neck (3 DoF), upper back (1 DoF), waist (2 DoF), left and right shoulder (3 DoF), elbow (2 DoF), wrist (1 DoF), hip (3 DoF), knee (2 DoF) and ankle (1 DoF). Additionally, we denote *C* for the input data, which consists of the depth maps, *D*, binary silhouette images, *S*, and RGB images, *R*. Because the input depth maps are depth values on the 2D plane, we can project them onto the world coordinate with the calibration parameters. We define a 3D point in the world coordinate as **p**. For notational brevity, we define the 3D point observed by the sensor as p*, and we define the 3D point generated based on the pose parameters, **q**, as **p**(**q**).

### 3.2. Pose Tracking

The core of our pose-tracking module follows the model registration algorithm in which the pose parameter, **q** ∈ *R*^36^, can be solved as a MAP problem [22]. Let *C_i_* be the input data at the current frame, *i*, which is composed of a depth map, *D_i_*, a binary silhouette image, *S_i_*, and an RGB image, *R_i_*. In addition, by denoting the previously reconstructed poses as *Q_m_*, the MAP problem can be formulated as:

(1) $$\arg\underset{\text{q}}{\max}\underset{\text{Likelihood}}{\underset{︸}{Pr(C|\text{q})}}\underset{\text{Prior}}{\underset{︸}{Pr(\text{q}|Q_{m})}}$$

We formulate the likelihood term and the prior term similar to Wei *et al.* [13]. However, Wei *et al.* [13] use a cylinder-like model to track human motion, while we use the SCAPE model for tracking. There is a large difference in the roll motion between these two models, e.g., when the arm rotates about the longitudinal axis, there is no difference in the cylinder-like model, while the SCAPE model shows a significant change. Obviously, the SCAPE model is closer to the motion truth than the cylinder-like model. In our practice, we add the RGB image term to track the motion of the roll. Additionally, because the SCAPE model has some details of the human body that are different from the observed depth map, we formulate a robust silhouette term to handle those differences.

#### 3.2.1. RGB Image Term

Depth maps can hardly reveal the motion of roll, so we attempt to find cues from the RGB images. Let *R_i_* and *D_i_* be the observed RGB image and depth map for the current frame, and let *R_i_*_-1_ and *D_i_*_-1_ be the observed RGB image and depth map for the last frame. First, we find and match the key points on *R_i_* and *R_i_*_-1_ using the ORBalgorithm [23]. Second, the matched key points are bound to the depth map of the same frame. For building the correspondence between the RGB key points and depth points, we project the depth map to the RGB image, and we find the nearest depth points to be the correspondence of the RGB points. However, there could be some mismatched correspondence between the RGB image and the depth map. To ensure the robustness of the RGB image term, we should remove the mismatches from the correspondence set. In our practice, we adopt an effective method for removing the mismatches. We simply calculate the distance of the pixels between the RGB key points and the depth points projected onto the RGB image. When the distance is beyond a threshold, we can consider the correspondence to be a mismatch, and we remove it from the correspondence set. Empirically, we set the threshold to three pixels. After we build the correspondence between the RGB image and the depth map, we can build the correspondence between successive depth maps according to the RGB matches. We define the points on the current observed depth as ${\text{P}}_{\text{rgb}}^{*}$, and we define the points transformed from the observed depth of the last frame by pose parameters, **q**, as **p***_rgb_*(**q**). Then, the RGB image term can be formulated as:

(2) $$E_{\text{rgb}}=\frac{{\left\|{\text{p}}_{\text{rgb}}(\text{q})-{\text{p}}_{\text{rgb}}^{*}\right\|}^{2}}{2\sigma_{\text{rgb}}^{2}}$$

**Figure 3.**
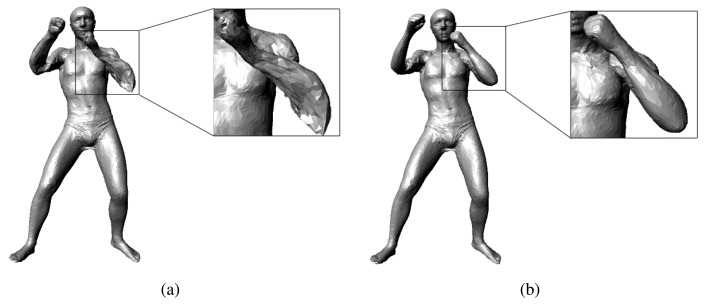
Impact of our RGB term: (**a**) shows the result without our RGB term; (**b**) shows the result with our RGB term.

Note that the RGB image term has the same form as the extra term in the MAP formula; we can optimize this term in the same way that we optimize the extra term in Equation (4). When optimizing the RGB image term, we should know the bones that the depth points belong to. Instead of binding a depth point to its nearest SCAPE model point, we bind it to the nearest bone. In this way, we can flexibly perform the transformation on the depth points. Figure 3 shows the impact of our RGB image term: the pose tracking without our RGB image term always gives a poor estimate of the roll motion of the limbs and head, while the pose tracking with the RGB image term can give accurate results.

#### 3.2.2. Silhouette Term

**Figure 4.**
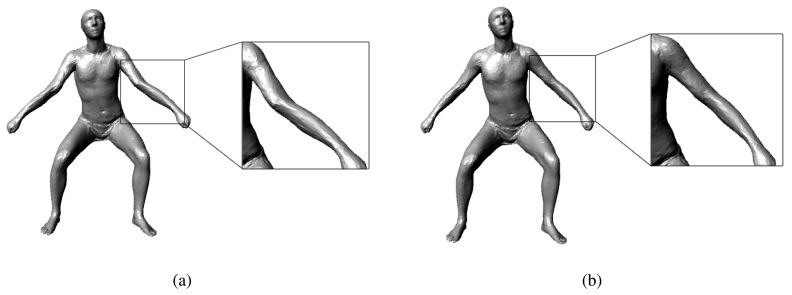
Impact of our silhouette term: (**a**) shows the result without our silhouette term; (**b**) shows the result with our silhouette term.

Note that the details of the SCAPE model, especially the silhouette, might not match the observed depth perfectly; we cannot evaluate the silhouette term in the same way as the depth image term. We should find a robust way to build the correspondence explicitly or implicitly between the rendered and the observed silhouette images. In our practice, we adopt the Coherent Point Drift (CPD) algorithm [24] on the 2D image space to build the correspondence for the silhouette images. The CPD algorithm can implicitly reject the mismatch that results from the details of the SCAPE model being different from the observed depth. Then, the silhouette term can be formulated as:

(3) $$E_{\text{silhouette}}=\frac{{\left\|S_{\text{render}}(\text{p}(\text{q}))-S\right\|}^{2}}{2\sigma_{\text{silhouette}}^{2}}$$

where *S_render_* represents the points on the silhouette image that are rendered from the SCAPE model, **p**(**q**) represents the points on the depth map that are rendered from the SCAPE model with the pose parameter, **q**, and *S* represents the points on the observed silhouette image. Figure 4 shows the impact of our new silhouette term: the pose tracking module without our silhouette term always twists the limbs of the SCAPE model improperly to match the details on the depth map, while the pose tracking with our silhouette term can give appropriate results.

#### 3.2.3. Optimization

After adding the RGB image term and the silhouette term, we can describe the pose-tracking problem as:

(4) $$\arg\underset{\text{q}}{\min}E_{\text{depth}}+E_{\text{extra}}+E_{\text{silhouette}}+E_{\text{rgb}}+E_{\text{prior}}$$

where:

(5) $$E_{\text{depth}}=\frac{{\left\|D_{\text{render}}(\text{x}(\text{q}),\text{q})-D\right\|}^{2}}{2\sigma_{\text{depth}}^{2}}$$

(6) $$E_{\text{extra}}=\frac{{\left\|\text{p}(\text{q})-{\text{p}}^{*}\right\|}^{2}}{2\sigma_{\text{extra}}^{2}}$$

(7) $$E_{\text{prior}}=\frac{{\left\|{\text{q}}_{i}-2{\tilde{\text{q}}}_{i-1}+{\tilde{\text{q}}}_{i-2}\right\|}^{2}}{2\sigma_{s}^{2}}$$

In the upper equations, *D_render_* represents the depth map rendered from the SCAPE model, *D* represents the observed depth map, **p**(**q**) represents the point on the SCAPE model, **x**(**q**) represents the coordinate of the depth pixel on the depth image and p* represents the point from the observed depth map. Instead of finding explicit correspondence using the traditional registration algorithm, we register the SCAPE models to the observed depth on the 2D plane. We project the 3D SCAPE model to the depth domain to render a hypothesized depth map, and we compare the rendered depth map with the observed depth map. For the overlapping region, if the difference at the same pixel between the rendered depth map and the observed depth map is no more than 6 cm, then we put this pixel into the correspondence set for the depth term, *E_depth_*, of our energy function. For the non-overlapping region, we search the closest points as correspondence for the extra term, *E_extra_*, of our energy function. To remove the outliers from the extra term, we set a depth difference threshold of 6 cm for the correspondence points. For the prior term, we assume that the current frame's velocity of the pose parameters should be close to the last frame's; as a result, a sudden change in the velocity is penalized in our energy function. Using the extended Lucas-Kanade algorithm [25], the above non-linear least square problem can be solved iteratively via linear system solvers. Performing a first-order Taylor expansion, we can obtain the following equations on *δ***q**:

(8) $$\frac{1}{\sigma_{\text{depth}}}(\nabla D_{\text{render}}\frac{\partial \text{x}}{\partial \text{p}}+\frac{\partial D_{\text{render}}}{\partial \text{p}})\frac{\partial \text{p}}{\partial \text{q}}\delta \text{q}=\frac{1}{\sigma_{\text{depth}}}(D-D_{\text{render}})$$

(9) $$\frac{1}{\sigma_{\text{silhouette}}}\nabla S_{\text{render}}\frac{\partial x}{\partial \text{p}}\frac{\partial \text{p}}{\partial \text{q}}\delta \text{q}=\frac{1}{\sigma_{\text{silhouette}}}(S-S_{\text{render}})$$

(10) $$\frac{1}{\sigma_{\text{extra}}}\frac{\partial \text{p}}{\partial \text{q}}\delta \text{q}=\frac{1}{\sigma_{\text{extra}}}({\text{p}}^{*}-\text{p})$$

(11) $$\frac{1}{\sigma_{\text{rgb}}}\frac{\partial {\text{p}}_{\text{rgb}}(\text{q})}{\partial \text{q}}\delta \text{q}=\frac{1}{\sigma_{\text{rgb}}}({\text{p}}_{\text{rgb}}^{*}-\text{p})$$

(12) $$\frac{1}{\sigma_{s}}\delta \text{q}=\frac{1}{\sigma_{s}}(2{\tilde{\text{q}}}_{\text{i}-1}-{\tilde{\text{q}}}_{\text{i}-2}-{\text{q}}_{\text{i}})$$

where the standard deviations, *σ_depth_*, *σ_silhouette_*, *σ_extra_*, *σ_rgb_* and *σ_s_*, are used to control the weights for each term and can be experimentally set to 1, 50, 0.3, 0.05 and 12.33.

Another problem in the optimization process is calculating the derivative, $\frac{\partial \text{p}}{\partial \text{q}}$. Directly calculating the derivative from the SCAPE model is difficult and time-consuming. To simplify this procedure, we use the rigid kinematics to approximate the derivative. The pose parameters, **q** = {*t_x_, t_y_, t_z_, θ*_0_*ξ̂, θ*_1_,…, *θ_n_*}, can be represented using the twist and exponential map parameterization [26,27]. Then, a point on the model can be formulated as:

(13) $${\text{p}}_{\text{i}}(\text{q})=T_{g}(t_{x},t_{y},t_{z})\sum_{m=1}^{n}[\omega_{i}^{m}\prod_{j=0}^{j_{m}}R_{g}(\theta_{\psi_{m}(j)}\;{\hat{\xi }}_{\psi_{m}(j)})]{\text{p}}_{\text{i}}$$

In the upper equation, *T_g_*(*t_x_, t_y_, t_z_*) represents the global transformation matrix, $\omega_{i}^{m}=1$, when the vertex belongs to the bone, *m*, and $\omega_{i}^{m}=0$, when the vertex does not belong to the bone, *m.* Furthermore, *j_m_* is the number of joints from the root to the bone, *m*, on the kinematic chain, and *ψ_m_*(*j*) maps the joint index to the global joint index. If we know the twist, *θξ̂*, and a point, **x_0_**, on the rotation axis, then the rotation matrix in the global coordinate can be represented as:

(14) $$R_{g}(\theta \hat{\xi })=\left(\begin{array}{cc} e^{\theta \hat{\xi }} & (I-e^{\theta \hat{\xi }}){\text{x}}_{0}\\ 0 & 1 \end{array}\right)$$

Because the pose parameter, **q**, that we use here describes the small relative motion between two successive frames, we can use the first-order Taylor expansion to approximate the exponential map:

(15) $$e^{\theta \hat{\xi }}\approx I+\theta \hat{\xi }$$

Then, the rotation matrix mapping in Equation (14) can be re-written as:

(16) $$R_{g}(\theta \hat{\xi })=\left(\begin{array}{cc} I+\theta \hat{\xi } & -\theta \hat{\xi }{\text{x}}_{0}\\ 0 & 1 \end{array}\right)$$

Consider a vertex, **p_i_**, on the bone, *m*, and a joint, *ψ_m_*(*k*), on the kinematic chain of bone *m.* Denote ${\text{x}}_{0}^{\text{k}}$ as a point on the rotation axis of the joint, *ψ_m_*(*k*), and let:

(17) $$\left(\begin{array}{c} {\text{x}}_{i}^{\text{k}}\\ y_{i}^{k}\\ z_{i}^{k}\\ 1 \end{array}\right)=\{\prod_{j=k+1}^{j_{m}}[R_{g}(\theta_{\psi_{m}(j)}\;{\hat{\xi }}_{\psi_{m}(j)})]{\text{p}}_{\text{i}}\}-{\text{x}}_{0}^{\text{k}}$$

(18) $$R_{g}^{k}=\prod_{j=1}^{k-1}\left[R_{g}(\theta_{\psi_{m}(j)}\;{\hat{\xi }}_{\psi_{m}(j)})\right]$$

Then, the derivative can be represented as:

(19) $$\frac{\partial {\text{p}}_{\text{i}}(\text{q})}{\partial \text{q}}=R_{g}^{k}(1:3,1:3)\left(\begin{array}{ccc} 0 & z_{i}^{k} & -y_{i}^{k}\\ -z_{i}^{k} & 0 & x_{i}^{k}\\ y_{i}^{k} & -x_{i}^{k} & 0 \end{array}\right))$$

In the upper equation, $R_{g}^{k}(1:3,1:3)$ represents the left upper 3 × 3 matrix in $R_{g}^{k}$.

### 3.3. Failure Detection

After the pose-tracking module, we build a failure-detection module to automatically detect failed pose-tracking results. The failure-detection module judges a failed pose-tracking instance by using the proportion of the unexplained area to the right matched area on the depth map. We project the model rendered from the tracked pose parameters to the observed depth map, and we define the right matched area as the overlapping area where the difference between the rendered pixel and the observed pixel is no more than 6 *cm.* Additionally, we define the unexplained area as the following three cases:

- The pixels belong to the observed depth map, but do not belong to the rendered depth map;
- The pixels belong to the rendered depth map, but do not belong to the observed depth map;
- The pixels overlap where the difference between the observed map and the rendered map is more than 6 *cm.*

When the proportion is more than 15%, we consider this pose tracking result to be a failed pose tracking.

## 4. Body Measurement

In this section, first, we use the first five frames to initialize a rough shape parameter for the SCAPE model. Although the initial shape parameter cannot be very accurate, it can be a baseline for the subsequent steps. Afterward, for each frame, a SCAPE model is optimized using the depth map at that time. Then, we transform all of these models into the template pose (the T-pose in Figure 2a) using the inverse Linear Blending Skinning (LBS) model. We weigh every vertex in these models of the template pose according to the z-direction error property of the Kinect and whether the vertex is in front of the sensor when the model it belongs to is in its original pose. A spatial-temporal average template model can be recovered through all of the frames that we captured. Based on the spatial-temporal model, we use the cue from time-space analysis to mitigate the effect of the clothes. Lastly, accurate human body parameters can be measured from this reconstructed model.

### 4.1. Shape Parameter Initialization

Before accurately reconstructing the human model, we must estimate a rough shape parameter as the baseline. In addition, the shape parameter can be used in initializing a SCAPE model to track the human motion. In our system, we use the first five frames of the sequence to solve the shape parameter. The process of generating a SCAPE model can be described as a problem of minimizing the objective function as [14]:

(20) $$E_{H}(Y)=\sum_{k}\sum_{j=2,3}{\left\|R_{k}\bar{{\left(U\beta +\mu \right)}_{k}}Q{\hat{v}}_{j,k}-(y_{j,k}-y_{1,k})\right\|}^{2}$$

where *R_k_* represents the rotation matrix for the triangle, *k*, on the SCAPE mesh, *β* represents the shape parameter, *U* and *μ* represent the pre-learned shape constants of the SCAPE model, *Q* represents the deformation matrix rendered from the relative rotation, *y_j,k_, y*_1,_*_k_* represent the vertices on the target model and *v̂_j,k_* represents the differential vertices on the template model. Here, *v̂_j,k_* can be calculated using the difference between a certain point and the start point on the same triangular face.

**Figure 5.**
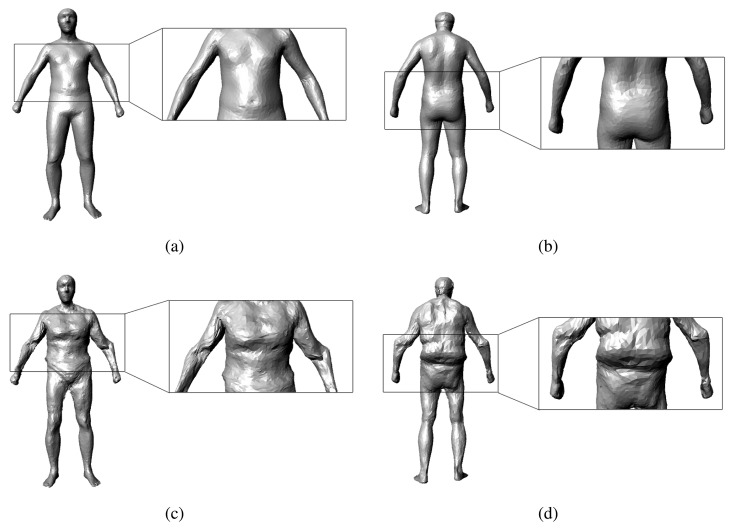
Necessity of our constraint of consistency: (**a**),(**b**) show the results with the constraint of consistency and (**c**),(**d**) show the results without the constraint of consistency.

When we estimate the shape parameter, the pose parameter can be seen as a constant vector. Therefore, the process of generating the SCAPE model can be reformulated as:

(21) Y=Aβ

where *A* is a matrix that is generated from the original template model and the constant pose parameter. As a partial completion problem, we can formulate the estimation of shape parameter *β* as:

(22) $$\arg\underset{\beta }{\min}{\left\|SA\beta -C\right\|}^{2}$$

where *S* is a matrix to select the points in the correspondence from the SCAPE model and *C* is the matrix of correspondence in the observed depth map. In our motion capture framework, we use the Coherent Point Drift (CPD) algorithm [24] to establish the correspondence between the SCAPE model and the observed depth map. However, because the SCAPE model is an over-fitting model of the shape parameter, if we solve Equation (22) directly, it could lead to an abnormal model (Figure 5). To address this issue, we account for the consistency of the shape parameters:

(23) $$\arg\underset{\beta }{\min}({\left\|SA\beta -C\right\|}^{2}+w{\left\|\beta -\beta_{\text{average}}\right\|}^{2})$$

In the upper equation, *β_average_* is a shape parameter we derive from a regularized shape model. Here, *w* is a factor of damp, and it can be decided through a synthetic-analytic procedure. First, we select a shape parameter from our database and adopt this parameter to generate a virtual depth map. Second, because we have already had the result of the shape parameter, we can inversely calculate the weight parameter. Of course, we should calculate the weight under the different conditions of human shapes. Thus, we use a least-squares solution to calculate the final weight parameter from multiple simulations. In the following section of the experiment, we set the weight parameter to 0.00015.

### 4.2. Spatial-Temporal Average Model Reconstruction

Because the Kinect sensor captures a partial view of an object, every frame from it can provide us with only 2.5D information. To recover the complete 3D information, we should synthesize the 2.5D information from different views along the time axis. At every frame, we attach the 2.5D information to the SCAPE model using the uniform Laplacian Coordinate [28]:

(24) $$\arg\underset{v}{\min}({\left\|Lv-Lv^{*}\right\|}^{2}+w{\left\|Sv-C\right\|}^{2})$$

In the upper equation, *v* are the result vertices from the deformation, v* are the original vertices on the baseline SCAPE model, *L* represents the uniform Laplacian matrix, *S* is a matrix to select the correspondence from the point set, *v*, and C is the correspondence from the depth map. As in Section 4.1, we establish this correspondence using the CPD algorithm. Usually, to capture the details from the depth, the factor of the weight, *w*, is set to no less than one. However, in our application, we want to avoid the interference of noise to obtain a more accurate measurement; thus, we set *w* to 0.25.

**Figure 6.**
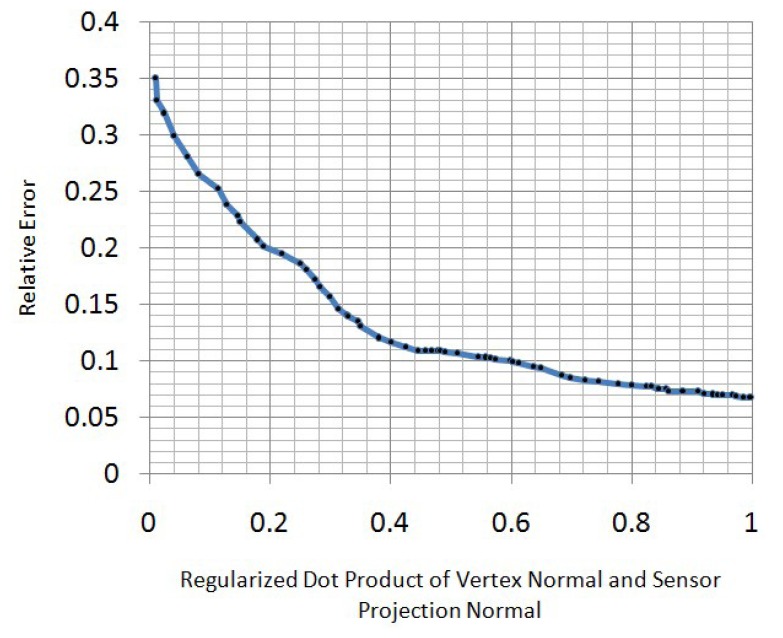
The relationship between z-direction relative measurement error and the regularized dot product of the vertex normal and sensor projection normal.

When we obtain the optimized models at every frame, we transform them into template pose using the inverse LBSmodel. Then, we weigh every vertex in these models to obtain a spatial-temporal average model. To decide the weight function here, the property of the Kinect z-direction error should be understood. In real applications, we find that the z-direction measurement error of a Kinect sensor increases when the regularized dot product of the vertex normal and sensor projection normal decreases (Figure 6). To best relieve the effect of this measurement error, we use a Gaussian function to re-weigh the vertex in front of the Kinect sensor:

(25) $$G(n)=e^{-\frac{{\left(n-1\right)}^{2}}{2\sigma^{2}}}$$

In the upper equation, *n* is the regularized dot product of the vertex normal and sensor projection normal, *i.e.*, *n* is the cosine of the angle between the sensor projection direction and the vertex normal direction. To ensure that *n* = 0 is the 3*σ* point of our weight function, we set the standard deviation, *σ*, to $\frac{1}{3}$. To allow for more information from the points that are not in front of the sensor, we stop decreasing the weight function when *n* ≤ 0. Instead, we set the weight to a constant when *n* ≤ 0. The constant is selected as 0.01, which is the approximation to the 3*σ* point of the Gaussian weight function. To summarize, the upper process of synthesizing optimized models on the time axis can be described as:

(26) $$v_{\text{average}}\;(j)=\frac{1}{\sum_{t=1}^{N}w_{t}^{j}}\sum_{t=1}^{N}w_{t}^{j}v_{t}(j)$$

where:

(27) $$w_{t}^{\text{j}}=\{\begin{array}{ll} 0.01 & \text{if vertex}\;j\;\text{is not in front of the sensor at time}\;t\\ G(n_{t}^{j}) & \text{if vertex}\;j\;\text{is in front of the sensor at time}\;t \end{array}$$

In the upper equation, $n_{t}^{j}$ is the regularized dot product of the vertex normal and the sensor projection normal.

### 4.3. Mitigation of the Effect of Clothes

**Figure 7.**
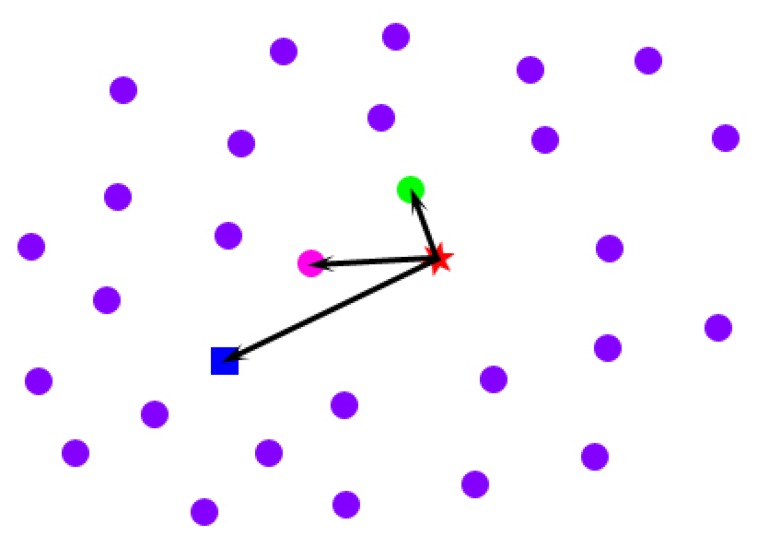
Time-space analysis to mitigate the effect of clothes: the red star represents a point on the model after shape parameter initialization (baseline). The blue rectangle represents the same point on the spatial-temporal average model. The pink, green and purple points are the same points on the optimized models of the T-pose across the frames. As described in Equation (28), the pink point is the best candidate for clothes effect mitigation.

Imagine the following situations: when a person extends his body, his clothes tend to be far away from his body; in contrast, when a person huddles up, his clothes tend to be close to his body. The spatial-temporal average model that we obtain in Section 4.2 from multiple poses is equivalent to the intermediate state between the above two situations. To mitigate the effect of clothes on the spatial-temporal average model, we conduct a space-time analysis for every point across the frames in which the point is in front of the Kinect sensor. For a specific point on the spatial-temporal average model (the blue rectangle in Figure 7), first, we pick all of its corresponding locations across the frames in which it is in front of the sensor (the pink, green and purple points in Figure 7). Second, we calculate the drifted vectors of these points from the original point (the red star in Figure 7) on the template model initialized in Section 4.1. To avoid the interference of the outliers that have relatively large errors in direction, instead of only finding a point with the drifted vector that has the least length (the green point in Figure 7), we select the point that not only has a relatively short drifted vector from the original point, but also has a drifted direction that is close to the spatial-temporal average point's drifted direction, as the best candidate (the pink point in Figure 7). In conclusion, we select a point across the frames as the best candidate for the mitigation of the effect of clothes using the following objective function:

(28) $$\arg\underset{\text{p}}{\min}[\left\|\text{p}-{\text{p}}_{\text{origin}}\right\|-w<({\text{p}}_{\text{aver}}-{\text{p}}_{\text{origin}}),\frac{\left(\text{p}-{\text{p}}_{\text{origin}}\right)}{\left\|\text{p}-{\text{p}}_{\text{origin}}\right\|}>]$$

**Figure 8.**
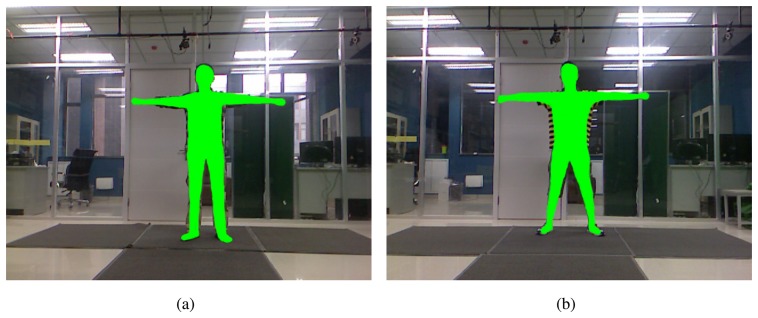
Results for the mitigation of the clothes: the green domains are the models projected on the RGB images. (**a**) shows the result of a person with relatively tight clothes; (**b**) shows the result of a person with more loose clothes than (**a**).

In the upper equation, **p** is one of the locations of a specific point across the frames, **p**_aver_ is the same point on the spatial-temporal average model, **p**_origin_ is the same point on the model after shape parameter initialization, < , > represents the dot product of two vectors and *w* is a trade-off factor between the least length and the direction constraint. Empirically, we set it to 0.1. Because of the occlusion, not every point can have an optimized location for the mitigation of the effect of clothes after the above-described process. We use the uniform Laplacian Coordinate [28] to obtain a consistent model:

(29) $$\arg\underset{v}{\min}({\left\|Lv-Lv_{\text{aver}}\right\|}^{2}+{\left\|Sv-C\right\|}^{2})$$

where *v* is the output vertices of the model, *v_aver_* is the input vertices of the spatial-temporal average model, *S* is the select matrix to select vertices that have an optimized location from the total vertices and *C* is a matrix that is composed of the optimized locations for the mitigation of the effect of clothes. Figure 8 shows the result of the mitigation of clothes compared with the related RGB images. More detailed results of this step and a numerical comparison of the accuracy between the spatial-temporal average model and the model after the mitigation of the clothes effect can be seen in Section 5.

### 4.4. Automatic Measurement

**Figure 9.**
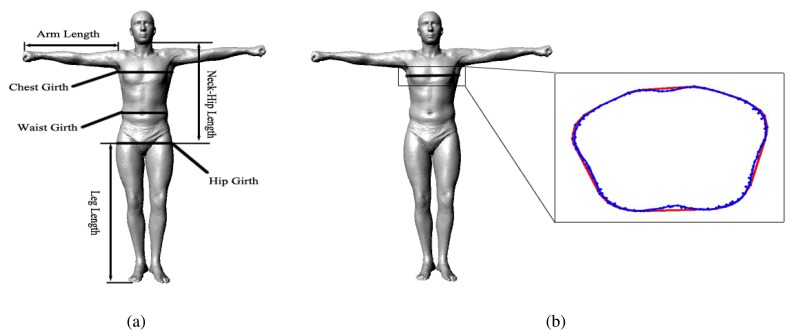
Automatic Measurement: (**a**) the human body parameters measured in our system; (**b**) we measure the circumference parameters using the girth of the convex hull (red curve) instead of the geodesic distance (blue curve), as we do in daily life.

For measuring the arm length, neck-hip length and leg length, we specify the indices of the start and end points according to the bone segment in advance, and the system can automatically measure these parameters from an obtained model. For measuring the chest girth, waist girth and hip girth, we specify the indices of a circle of points around a corresponding location according to the standard definition in advance; afterward, our system constructs a convex hull from these points during runtime, and the parameters can be measured from the girth of the convex hull automatically by our system (Figure 9b). Figure 9a shows all of the human body parameters that our system measures.

## 5. Results and Discussion

**Figure 10.**
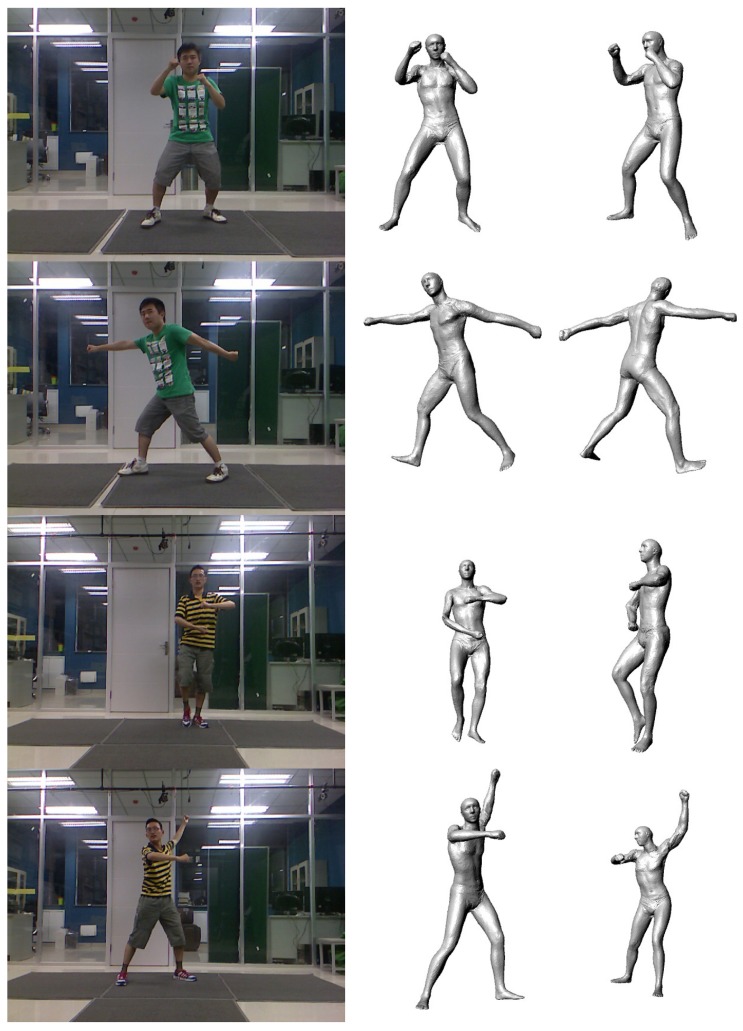
Results of the motion capture module: Column 1 shows the RGB images. Column 2 shows our results in the front view. Column 3 shows different viewpoints of column 2.

**Figure 11.**
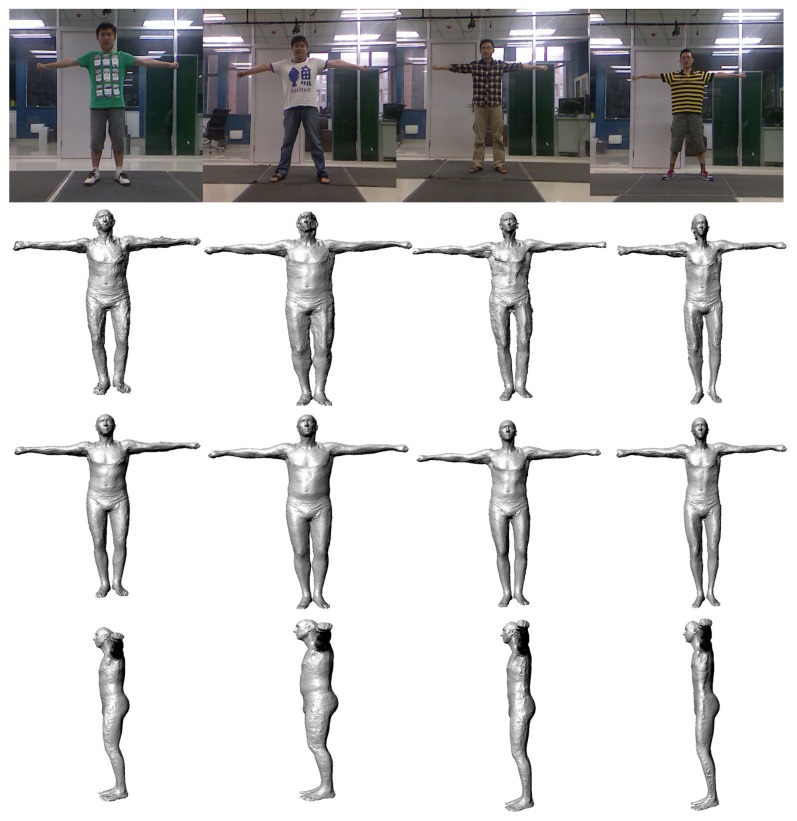
Results of the model reconstruction: Row 1 shows the RGB images. Row 2 shows the results of average models. Row 3 shows the results after mitigating the clothes effect. Row 4 shows a different viewpoint of row 3.

**Figure 12.**
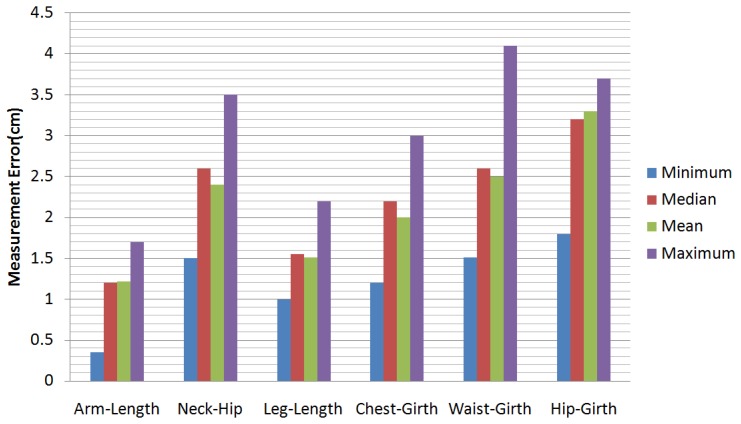
The statistics for the error of the proposed method.

In our experiments, we have tested a total of 55 sequences of video. There are 25 men and 10 women tested in our experiments (some people were tested more than once). The people measured in our experiments are 20 to 45 years old, their weights range from 40 kg to 88 kg and their heights range from 1.55 m to 1.90 m. Figure 10 shows some results of our motion capture module, and Figure 11 shows some results of our model reconstruction. The statistics of the mean, the median and the minimum and the maximum of the error of our proposed method can be seen in Figure 12. Specifically, without the pose recovery from failed pose tracking, the inaccuracy of the pose estimation affects the body parameter measurements to a large degree. Failed poses often lead to increased outliers in the subsequent procedure, which results in an increase in the average relative error of the body measurements from 2.62% to 25.8%. For this reason, we introduce a failure detection module to detect failed poses and to recover the system from failed pose estimation automatically. Table 1 shows the average computational time statistics for our system.

In the remaining part of this section, we will compare our method with the state-of-art methods [6,7]. We also compare our results after mitigation of the effect of clothes to the results before mitigation of the effect of clothes. These comparisons can explicitly show the superiority and effectiveness of our method in measuring the human body.

As a result, our proposed method can accurately measure the body parameters of dressed humans with large-scale motions. In other words, our proposed method can be easily applied in public situations, such as shopping malls, police stations and hospitals. Additionally, because the total price of our system is approximately $150, it can be widely used in home situations.

### 5.1. Quantitative Comparison with Home 3D Body Scans [6]

First, we compare our method to [6] for measuring almost naked people. In our experiment, we measure 25 different almost naked people to calculate the average error values, and we compare them to the reported results from [6], as shown in Table 2.

As can be seen in Table 2, although our method provides better results in most entries, there is no obvious superiority under the conditions of an almost naked body and no large-scale motion. To show our method's advantage, we compare our solution to the proposed method [6] for measuring people with relatively tight clothes. A comparison of average errors can be seen in Table 3 (the result of Weiss *et al.*'s proposed method comes from our implementation of [6]). Obviously, the method from [6] does not have as high a capacity for handling people wearing clothes as our system has. Their system cannot mitigate the effect of clothes.

### 5.2. Quantitative Comparison with KinectAvatar [7]

**Figure 13.**
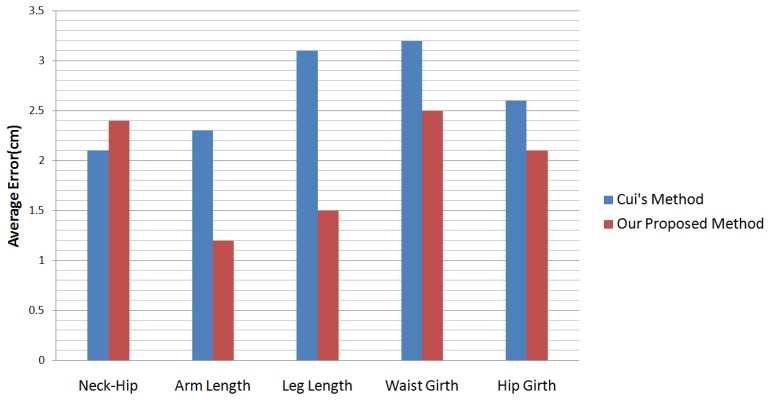
Comparison of the accuracy of measuring a human wearing clothes with KinectAvatar [7].

Cui *et al.* [7] presents a method that can capture the details of human bodies. They capture people with small-scale motion from no less than 12 views. For every view, they capture 30 ∼ 40 chunks. However, because their motion capture is limited to a small-scale motion, they cannot perform the best measurement on the human body statistically. A comparison between their method and ours can be seen in Figure 13.

In Table 3 and Figure 13, our proposed method provides slightly worse results only in the entry of the neck-hip length. The reason is that, in our setting, people can perform large-scale motion in front of the sensor, while in Cui *et al.*'s and Weiss *et al.*'s methods, people were limited to a relatively constant pose.

### 5.3. The Effectiveness of the Mitigation of Clothes Effect

Before evaluating our method's effectiveness, a parameter for measuring the tightness of the clothes should be defined. Of course, for different parts of the human body, the tightness of the same clothes could be different. Thus, we should evaluate the tightness of different parts of the clothes separately. Here, we use the variance of a human body parameter in different poses to evaluate the tightness of the clothes at the location related to the parameter:

(30) $$\sigma_{\text{looseness}}^{2}=\frac{1}{N}\sum_{i=1}^{N}{\left(\frac{l_{i}-\mu }{\mu }\right)}^{2}$$

In the upper equation, *l_i_* is the value of a certain human parameter in the *ith* pose, and *μ* represents the mean of this human parameter across all of the frames. For every *l_i_*, we divide it by *μ* to eliminate the effect of different human shapes.

**Figure 14.**
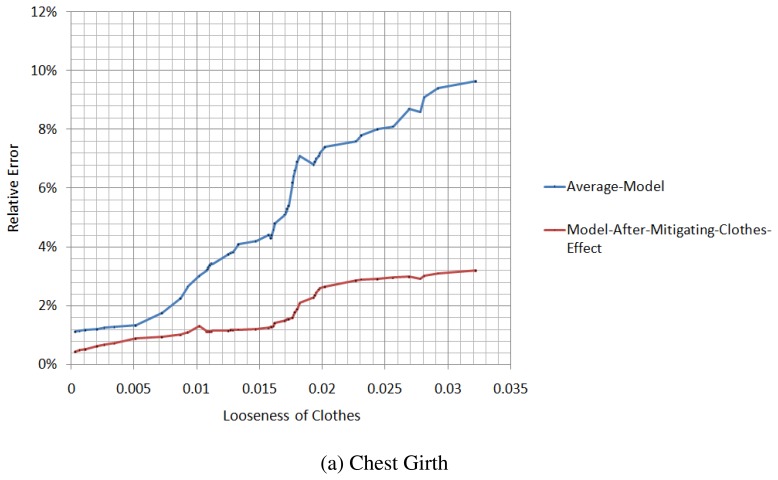

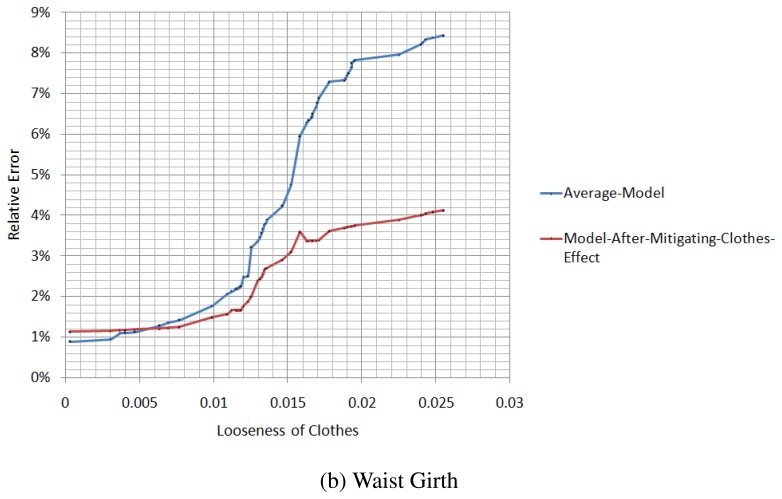
Comparison of the model after the mitigation of the clothes effect with the spatial-temporal average model.

We measure the relative errors of the chest girth and waist girth from the spatial-temporal average model and the model after the mitigation of the clothes effect, in different situations of tightness, as illustrated in Figure 14. When the looseness of the clothes is relatively small, the difference in the relative error between the average model and the model after the mitigation of the clothes effect is not large, and for some parameters, the accuracy of the average model is even better than the model after the mitigation of the clothes effect. However, with the increase of the looseness of the clothes, the relative error of the average model increases rapidly, while the relative error of the model after the mitigation of the clothes effect increases slowly.

## 6. Conclusions

In this paper, we present a novel approach that can measure the human body in clothes with large-scale motion accurately. The key contribution of our paper is to mine the cue from different poses in the temporal domain and the information from the spatial depth map to mitigate the effect of the clothes. Additionally, our reconstruction of the average model provides a robust estimate of the deformation direction from the original model to the model that is closest to a real human body. Another contribution of our paper is extending a motion capture framework from the cylinder-like model to the SCAPE model by using cues from the RGB images and the silhouette. Quantitative evaluations show that our solution for measuring parameters of the human body is more accurate than the present methods. Additionally, a comparison to the average model with large-scale motion shows that our method of mitigating the clothes effect is effective.

In the future, we will attempt to follow the core idea of Non-Rigid Structure from Motion and find a solution for measuring people with large-scale motion by using only an RGB camera.

## Figures and Tables

**Table 1.** Average computational time statistics for our system: the running time statistics were gathered from testing our implementation on a dual core 2.33 GHz Intel processor.

Procedure	Time Consuming
Pose Recovery	6.32 s per frame
Shape Parameter Recovery	55.2 s
Weighed-Average Model Recovery	5.45 s per frame
Mitigation of the Effect of Clothes	7.94 s per frame

**Table 2.** Comparison of the accuracy of almost bare human measurement with home 3D body scans [6].

	Arm Length	Chest Girth	Neck to Hip Distance	Hip Girth	Thigh Girth
Error of [6] (cm)	2.6	**1.5**	**2.3**	3.5	1.7
Error of ours (cm)	**1.7**	1.6	2.6	**1.9**	**1.3**

**Table 3.** Comparison of the accuracy of measuring a human wearing clothes with home 3D body scans [6].

	Arm Length	Chest Girth	Neck to Hip Distance	Hip Girth	Thigh Girth
Error of [6] (cm)	2.0	8.5	**2.3**	6.3	4.7
Error of ours (cm)	**1.2**	**2.2**	2.4	**2.1**	**1.5**
